# Dosimetric comparison of robust angles in carbon-ion radiation therapy for prostate cancer

**DOI:** 10.3389/fonc.2023.1054693

**Published:** 2023-02-16

**Authors:** Han-Back Shin, Changhwan Kim, Min Cheol Han, Chae-Seon Hong, Seyjoon Park, Woong Sub Koom, Jin Sung Kim

**Affiliations:** ^1^ Department of Radiation Oncology, Yonsei Cancer Center, Yonsei University College of Medicine, Seoul, Republic of Korea; ^2^ Department of Radiation Oncology, Yonsei Cancer Center, Yonsei Severance Hospital, Seoul, Republic of Korea

**Keywords:** prostate cancer, dosimetric study, carbon-ion radiation therapy, particle therapy, RBE-weighted dose, LET value

## Abstract

The objective of this study is to compare the plan robustness at various beam angles. Hence, the influence of the beam angles on robustness and linear energy transfer (LET) was evaluated in gantry-based carbon-ion radiation therapy (CIRT) for prostate cancer. 10 patients with prostate cancer were considered, and a total dose of 51.6 Gy (Relative biological effectiveness (RBE) was prescribed for the target volume in 12 fractions. Five beam field plans comprising two opposed fields with different angle pairs were characterized. Further, dose parameters were extracted, and the RBE-weighted dose and LET values for all angle pairs were compared. All plans considering the setup uncertainty satisfied the dose regimen. When a parallel beam pair was used for perturbed scenarios to take into account set-up uncertainty in the anterior direction, the LET clinical treatment volume (CTV) *D*_95%_ standard deviation was 1.5 times higher, and the standard deviation of RBE-weighted CTV *D*_95%_ was 7.9 times higher compared to an oblique pair. The oblique beam fields were superior in terms of dose sparing for the rectum compared to the dose distribution using two conventional lateral opposed fields for prostate cancer.

## 1 Introduction

Radiation therapy must deliver the prescribed dose to the target volume while avoiding excessive exposure to normal organs. Particle [e.g., proton (1), carbon-ion (2)] therapy (PT) provides sharper dose distributions compared to conventional X-ray therapy by utilising the Bragg peak (3). Moreover, compared to conventional radiation treatments, PT enables dose escalation which improves tumour control rates and minimizes radiation exposure to organs at risk (OAR) (4). Consequently, interest in prostate cancer treatment using PT is increasing (5).

The PT beam angle has a dominant effect on the patient dose distribution due to the limited number of beams used in PT. In general, beam angles should be set to avoid OARs. With prostate cancer, many PT facilities use bilateral horizontal angles to deliver conformal dose distributions for the target volume and to avoid high-dose distributions to the OARs, such as the bladder and rectum.

Clinically, PT for the prostate has been conventionally performed using a fixed beam nozzle with bi-lateral angles. PT with various beam angles using gantries could provide a more robust dose distribution than a fixed beam. Several studies on beam angle optimization of gantry-based PT treatment for prostate cancer have been published. Tang et al. performed a planning study for prostate cancer according to the variation of the treatment beam angle with the gantry in proton therapy (6). Kubota et al. evaluated the influence of the range and setup uncertainties of carbon-ion radiation therapy (CIRT) for prostate cancer with fixed fields at 0° and 90° and a rotating patient couch (7). Moteabbed et al. created an anterior-oblique plan for prostate cancers by passive scattering protons, and the planned dose distribution was compared with that of a conventional bi-lateral plan (8). Unfortunately, the robustness of dose distribution according to the beam angle for range and setup uncertainties in CIRT for prostate cancer has not yet been evaluated.

Determining CIRT robustness requires careful evaluation of the linear energy transfer (LET) distribution because of its high relative biological effectiveness (RBE). The LET distribution is affected by the incident beam field angle and path and has unique characteristics in that the value increases toward the end of the primary particle range (9, 10). This LET characteristic is advantageous for ion beams, and it has been experimentally proven that the RBE value increases with increasing LET value (11, 12). The three applied RBE models (mixed beam model, microdosimetric kinetic model (MKM), and local effect model (LEM)) reflect the effect of LET by converting the absorbed carbon ion distribution into an RBE-weighted dose distribution (13–16). Previous studies that used Monte Carlo calculation for obtaining the LET distribution have been performed (10, 17); however, the possibility of optimization using LET has been raised, and it has not been used clinically. Recently, dose-average LET calculation and visualization have been realised using RayStation 11B version, a commercial treatment planning system (TPS) (18). Therefore, we compared RBE-weighted dose and LET values according to the beam angle configurations using RayStation 11B. A planning study of CIRT with a gantry for prostate cancer was performed using different beam angle configurations, and various dosimetric parameters obtained from the RBE-weighted dose and LET values were compared. These evaluations included setup uncertainties using a robust optimization technique.

## 2 Materials and methods

### 2.1 Patient

Data for 10 patients, who had undergone treatment for prostate cancer using tomotherapy at Yonsei Cancer Center, were retrospectively applied to simulate CIRT. This study was approved by the Institutional Review Board of Yonsei University Hospital (approval number: 4-2022-0502), and the patient records and information were anonymized prior to analysis. Ten patients in this study whose CT data is used in this study has previously received tomotherapy treatment. Computed tomography (CT) images were acquired within an hour of the start of the patient treatment session. The pixel resolution of scanned images was approximately 1.0 × 1.0 mm^2^, and the slice thickness of the images was fixed at 2.00 mm. All images were acquired using 16-slice CT scanners, Sensation Open (Siemens Healthineers, Erlangen, Germany) and Aquilion LB (Canon Medical Systems, Tokyo, Japan). **Table 1** summarizes the attributes of the ten prostate patients included in this study.

**Table 1:** Patient attributes for this study.

	Age	Stage	Gleason score	Prostate volume (cc)
1	74	T2	4 + 3 = 7	54.77
2	67	T2c	3 + 4 = 7	46.46
3	76	T2c	4 + 3 = 7	36.79
4	78	T2	3 + 3 = 6	25.39
5	76	T2a	3 + 3 = 6	26.52
6	79	T1/2	4 + 3 = 7	66.28
7	83	T3b	5 + 4 = 9	44.69
8	78	T1b	4 + 3 = 7	45.34
9	75	T2a	3 + 4 = 7	62.75
10	83	T4	5 + 4 = 9	95.42

### 2.2 Treatment planning strategy

Prostate plans for patients were calculated and optimized using a research version of the RayStation 11 B treatment planning system (RaySearch Laboratories AB, Stockholm, Sweden). For contouring structures of the prostate and rectum, empty the rectum and bladder 1 hour before CT simulation, then drink about 1,000 ml of water, and the CT simulation was taken in the supine position. The prostate and rectum were delineated by the physician based on the simulation CT and MR fusion images and then reviewed by the experienced physician. The rectum includes the lowest level of ischial tuberosities and the superiorly of the S3 level, leaving the presacral region. Depending on the patient, all of these regions were delineated as much as possible. The dose constraints and margins of the CIRT planning for prostate cancer patients were based on National Institute of Radiological Sciences (NIRS) protocols.

In this study, the dose constraint was considered to be the RBE-weighted dose. A CIRT dose regimen of 51.6 Gy (RBE) in 12 fractions was adopted for localized prostate treatment following previous CPal (18) and SScl (19) protocols (9904 (4) and 1002, respectively), and the prostate volume receiving >95% of the prescribed dose was >95%. Based on the protocols, the recommended dose constraint for the rectum was set to *V*_53Gy_ (RBE) = 0%, *V*_50Gy_ (RBE) ≤ 7%, and *V*_40Gy_ (RBE) ≤ 16%, and dose constraints for other OARs were not considered (20). Physical dose distributions were calculated using the pencil beam algorithm, and biological optimization was based on a modified version of the microdosimetric kinetic model (mMKM).

For plan optimization, single-field optimization (SFO) or multifield optimization (MFO) could be applied to particle therapy; the former optimised each irradiation field and the latter optimised all fields simultaneously (21, 22). Herein, MFO was applied for plan optimisation and compared with SFO for various RBE models, which indicated that MFO is the preferred technique for reducing RBE-weighted dose uncertainties arising from RBE model variations (23). Additionally, the delivered LET could be relatively lower in OAR (23). Planning using SFO is less sensitive to setup and range uncertainties; however, the LET value delivered to the OAR may increase.

**Figure 1:**
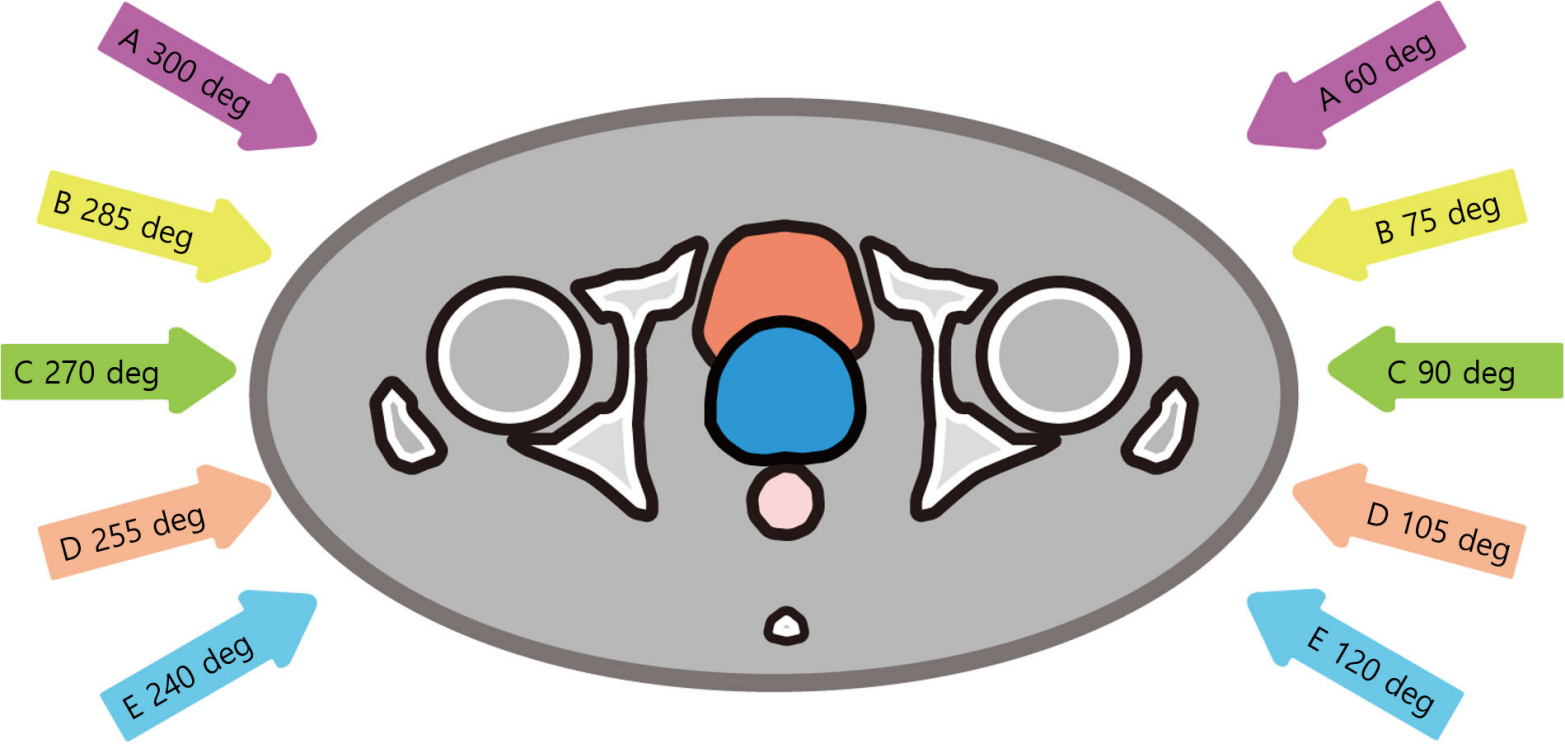
Beam angle configurations in this study.

A total of five plans comprising two opposed fields with different angle pairs (A: 60° and 300°, B: 75° and 285°, C: 90° and 270° (a.k.a. conventional two opposite lateral fields), D: 105° and 255°, and E: 120° and 240°) were generated for every patient. **Figure 1** illustrates the five beam angle configurations considered in this study.

To create robust plans for prostate treatment in CIRT, an optimization technique based on the clinical target volume (CTV) was implemented (24, 25). Note that the conventional margin-based plan for particle therapy, including CIRT, does not provide sufficient robustness against perturbed scenarios (24). In this study each plan was created by considering patient position uncertainties in six directions (anterior, posterior, cranial, caudal, and left and right laterals). The position uncertainty for robust margins based on each direction was set in accordance with the NIRS protocol (26) as follows:

- Anterior and two laterals: 10 mm
- Cranial, caudal, and posterior: 5 mm

The robust optimisation in RayStation is based on the minimax optimisation method (27). The minmax optimization function *f* considering scenarios in the set *S* having weight value *w*, which were formulated as an optimisation problem as Equation 1:


(1) $$\underset{x\in X}{\min}\frac{1}{\left|S\right|}\underset{s\in S}{\sum }\overset{n}{\underset{i=1}{\sum }}w_{i}f_{i}\left(d\left(x;s\right)\right)$$

Where *X* is the set of feasible variable, and *d(x;s)* is the dose distribution as a function of the variables *x* and scenario *s* (27).

A total of 18 scenarios were considered, which was the nominal setup error scenario without setup error and the case of shifting according to the degree of position uncertainty in the directions, it was modelled as displacement of the isocentre for an ellipsoid with radii.

### 2.3 Plan evaluation strategy

To evaluate the dosimetric effect of the prepared plans, a robustness evaluation was performed based on both the planned scenario and the perturbed scenarios, considering the margin in six directions (anterior and lateral: 10 mm; cranial, caudal, and posterior: 5 mm). The margin used in this study considered both range uncertainty and position uncertainty. Therefore, in the plan evaluation, range uncertainty and position uncertainty were considered and evaluated according to each direction and incident beam field. For robust evaluation of perturbed scenarios, a total of 300 scenarios were used considering 6 margin directions and 5 different angles (30 scenarios/patient). For instance, in the perturbed scenario for the anterior direction, a 10 mm setup uncertainty in the anterior direction based on each angle configuration is considered.

### 2.4 Quantitative analysis parameters for REB-weighted dose and LET

This study compared various dosimetric parameters among CIRT-based prostate plans according to the beam angles. The RBE-weighted dose distributions for the prostate and rectum were calculated and compared with those of each plan. For this purpose, the dose volume histogram (DVH) indices for the clinical target volume (CTV), that is, mean dose (*D*_mean_), the dose covering 90% of the volume (=*D*_90%_), the dose covering 99% of the volume (=*D*_99%_), *D*_95%_, and *D*_2%_ were compared. For the rectum, dose differences were compared by comparing *D*_mean_, *D*_2%_, *V*_53Gy_, *V*_50Gy,_ and *V*_40Gy_.

As an index for plan comparison, the dose-average LET value and RBE-weighted dose were compared. The LET distribution was analysed in the same way as the evaluation indices of the RBE-weighted dose. In the case of LET indices for the CTV and rectum, the same indices as those used for RBE-weighted dose indices, that is, the LET covering 90% of the volume (=*D*_90%_), *D*_mean_, *D*_99%_, *D*_95%_, and *D*_2%_ were compared. However, *V*_53Gy_, *V*_50Gy_, and *V*_40Gy_ were excluded from the evaluation indices because there were no prescribed LET values.

## 3 Results

### 3.1 Dosimetric comparison according to angle configurations with planned scenarios

**Figure 2:**
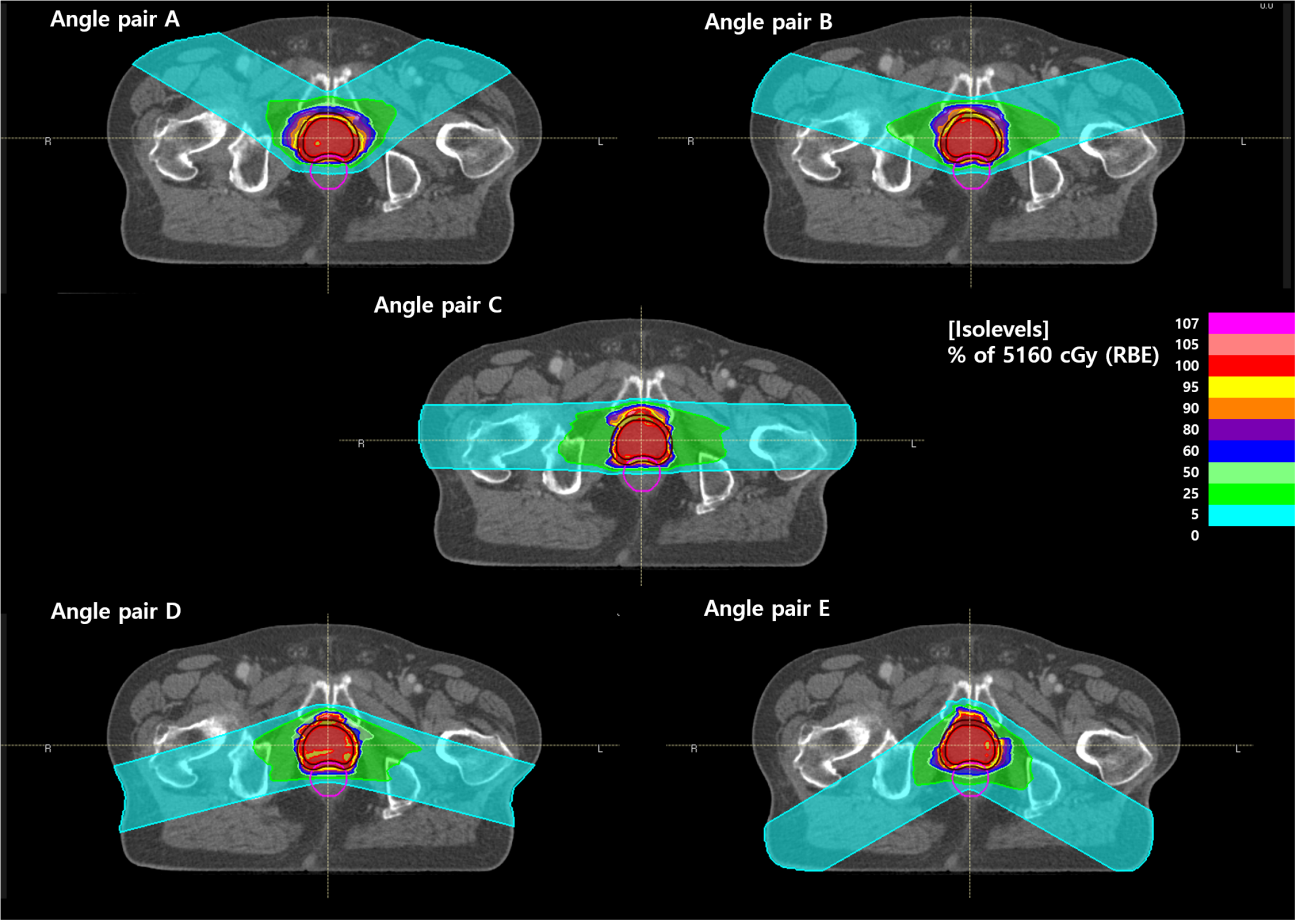
RBE-weighted dose distribution according to the angle pair **(A–E)**.

**Figure 3:**
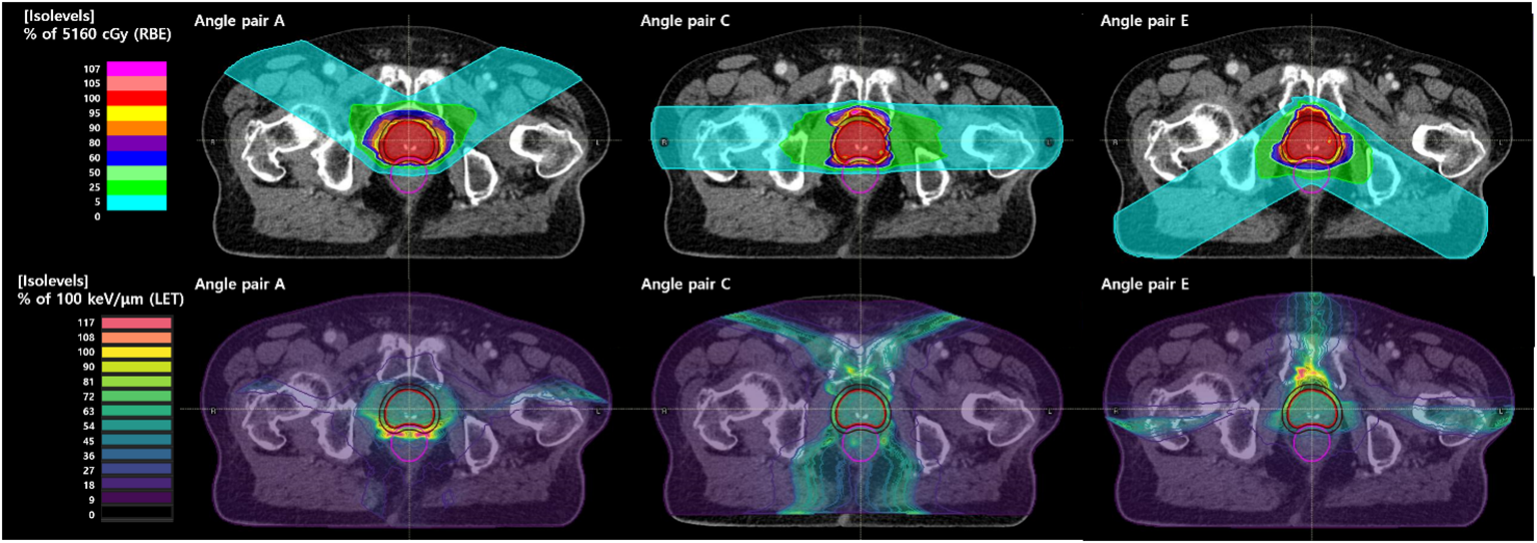
LET distributions of angle pair (A, C, E).

**Table 2** shows the nominal plan without robust evaluation results for 10 patients. The RBE and LET values according to the angle pairs are summarized for the dosimetric parameters of the prostate and rectum. As shown in **Table 2**, all RBE-weighted dose regimens were satisfied regardless of the angle configurations. For the RBE-weighted dose of prostate *D*_95%_, the difference was within 0.1%, because the scale was adjusted to satisfy the prescription dose. When compared with angle pair C in the RBE-weighted dose, the index showing the largest relative difference was the mean dose of the rectum, which was -8.6% for angle pair A and +26.1% for angle pair E. When the prostate *D*_2%_ of the LET value was compared with angle pair C, angle pair A and E showed differences of +15.6% and +4.6%, respectively. In *D*_2%_ of the rectum, the difference was +37.1% for angle pair A and 68.9% for angle pair E when compared with angle pair C. **Figure 2** shows the RBE-weighted dose distribution for each angle of a patient case, and **Figure 3** illustrates the LET distribution with angle pairs A, C, and E of the patient case, which is the same as that in **Figure 2**. From the point of view of the rectum, relatively high and low LET values in comparison with the conventional method were distributed in angle pairs A and E, respectively.

**Table 2:** Dosimetric parameters of the nominal plans using five different angles pair conditions including conventional two opposite lateral fields (90° & 270°).

Structure	Parameter	Angle pair				
		A (60°–300°)	B (75°–285°)	C (90°–270°)	D (105°–255°)	E (120°–240°)
		RBE (Gy)^*^/LET (keV/μm)	RBE (Gy)/LET (keV/μm)	RBE (Gy)/LET (keV/μm)	RBE (Gy)/LET (keV/μm)	RBE (Gy)/LET (keV/μm)
Prostate	mean dose	52.20 ± 0.06/ 53.3 ± 3.1	52.25 ± 0.08/ 50.9 ± 2.9	52.48 ± 0.11/ 51.2 ± 2.1	52.34 ± 0.08/ 50.9 ± 2.9	52.29 ± 0.07/ 51.7 ± 2.9
	*D*_90%_	51.73 ± 0.03/ 46.5 ± 2.5	51.75 ± 0.06/ 47.0 ± 2.4	51.83 ± 0.05/ 47.9 ± 1.9	51.78 ± 0.04/ 47.5 ± 2.2	51.76 ± 0.04/ 46.5 ± 2.6
	*D*_99%_	51.33 ± 0.08/ 44.5 ± 2.0	51.35 ± 0.12/ 45.9 ± 2.3	51.00 ± 0.39/ 46.7 ± 1.9	51.17 ± 0.14/ 46.0 ± 2.0	51.17 ± 0.17/ 44.5 ± 2.7
	*D*_95%_	51.60 ± 0.00/ 45.7 ± 2.3	51.62 ± 0.06/ 46.5 ± 2.3	51.60 ± 0.00/ 47.4 ± 1.9	51.60 ± 0.00/ 46.9 ± 2.1	51.60 ± 0.00/ 45.7 ± 2.6
	*D*_2%_	52.98 ± 0.09/ 69.6 ± 3.4	53.06 ± 0.16/ 60.0 ± 3.4	53.47 ± 0.28/ 58.8 ± 2.6	53.20 ± 0.13/ 58.4 ± 4.0	53.20 ± 0.19/ 61.6 ± 3.5
Rectum	mean dose	6.25 ± 1.87/ 41.2 ± 5.7	7.03 ± 1.68/ 61.8 ± 4.7	6.79 ± 1.50/ 49.3 ± 5.1	8.13 ± 1.49/ 41.3 ± 4.8	9.19 ± 1.72/ 20.5 ± 1.6
	*D*_2%_	51.74 ± 0.78/ 115.7 ± 7.3	51.92 ± 0.58/ 102.0 ± 4.6	51.50 ± 0.80/ 72.7 ± 8.3	52.13 ± 0.35/ 56.3 ± 5.1	48.17 ± 2.50/ 43.1 ± 6.0
	*V*_53Gy_**	0.4 ± 0.2	0.6 ± 0.5	0.5 ± 0.5	0.8 ± 0.6	0.2 ± 0.3
	*V*_50Gy_**	3.3 ± 0.9	3.3 ± 0.5	3.0 ± 0.6	3.4 ± 0.3	1.6 ± 0.9
	*V*_40Gy_**	6.1 ± 1.3	6.8 ± 0.9	6.8 ± 1.0	7.3 ± 0.6	2.6 ± 1.3

*RBE-weighted dose.

**For V_53Gy_, V_50Gy_, and V_40Gy_, only RBE-weighted dose values were indicated.

The relative biological effectiveness (RBE)-weighted dose and dose-averaged linear energy transfer (LET) value (mean ± standard deviation) are averaged over 10 cases according to the angle pairs.

### 3.2 Dosimetric comparison according to angle configurations with perturbed scenarios

**Figure 4:**
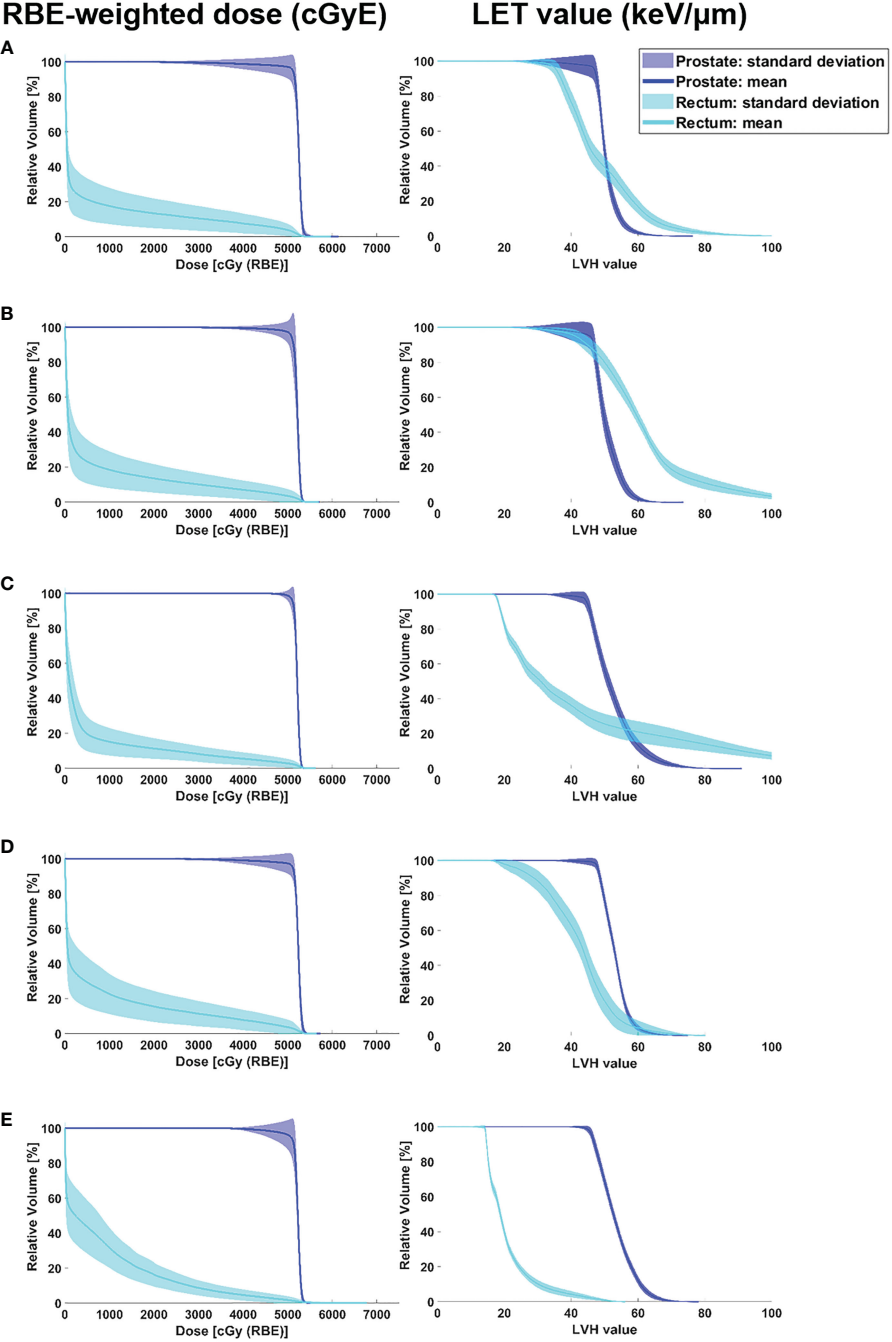
RBE-weighted dose volume histograms and LET volume histograms of the prostate and rectum according to angle pair A–E including data of robust evaluation result in all cases. The colour wash represents the standard deviation of DVH bands for dose distributions covering setup uncertainty for the CTV and rectum in the optimised plan, and the solid lines correspond to the DVHs and LVHs for the average value. **(A)** angle pair A, **(B)** angle pair B, **(C)** angle pair C, **(D)** angle pair D, and **(E)** angle pair E.

**Figure 4** shows the RBE-weighted dose-volume histogram (DVH) and LET volume histogram (LVH) considering perturbed scenarios for the prostate and rectum according to angle pairs. Based on the RBE-weighted dose, the pass rate of the CTV dose regimen was as follows: angle pair A, 33.3%; E, 20.0%; B and D, 6.7%; and C, 5.0%. The pass rate of the rectal dose regimen was as follows: angle pair E, 38.3%; B, 20.0%; A and D, 18.3%; and C, 16.7%. In the RBE-weighted DVH, the DVH distribution for the CTV mean dose was narrowest in angle pair C, followed by D, B, E, and A in that order. The mean rectal dose for RBE-weighted DVH was the narrowest in angle pair C, followed by E, B, A, and D. In the case of *D*_2%_ for CTV, which is expressed as a hot point, when compared with angle pair C, angle pair A was the highest at 101.1%, and angle pair D was the lowest at 99.41%.

**Figure 5:**
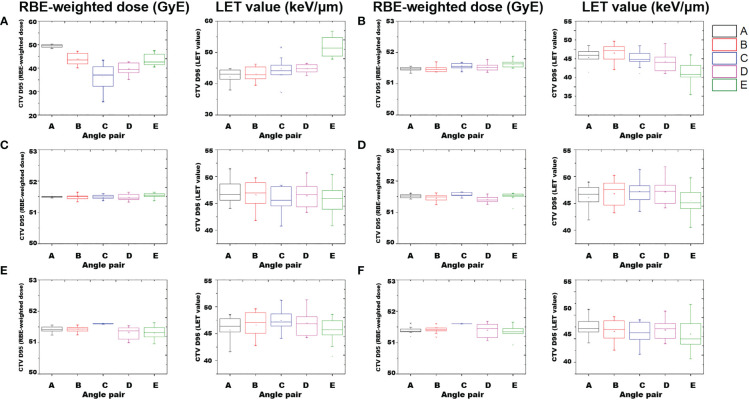
Box-plot of RBE-weighted dose and LET value of CTV *D*_95%_ according to angle pair A–E. Average and standard deviation, min, max values are indicated. Results of perturbed scenarios in 6 directions: **(A)** anterior, **(B)** posterior, **(C)** cranial, **(D)** caudal, **(E)** left, and **(F)** right.

From the LET volume histogram, the average value of CTV *D*_95%_ shows that the maximum difference between angle pairs A–E was 2.7%. The LET values were calculated using angle pairs in the following order E: 46.91 keV/μm, B: 46.55 keV/μm, D: 46.44 keV/μm, A: 46.28 keV/μm, and C: 45.63 keV/μm. For the LVH band evaluation of the mean CTV dose, angle pair A was the widest and angle pair E was the narrowest (A > C > D > B > E). For the CTV *D*_2%_, angle pair C showed the highest value at 72.56 keV/μm, and angle pair B showed the lowest value at 60.34 keV/μm (C > A > D > E > B). For the rectum mean dose, the average LET values were in the order of angle pair B: 62.00 keV/μm, A: 49.19 keV/μm, C: 41.78 keV/μm, D: 41.76 keV/μm, and E: 20.63 keV/μm. When the degree of LVH band distribution was quantified as the standard deviation of the rectum mean dose, angle pair C was the widest, followed by A, B, D, and E. In case of rectum *D*_2%_, angle pair C showed the highest value of 115.18 keV/μm, and angle pair E showed the lowest at 43.53 keV/μm (i.e., C > B > A > D > E). **Figure 5** shows a box-plot of RBE-weighted dose and LET value for CTV *D*_95%_ as a function of angle pair, indicating the average, standard deviation, min, and max values for each direction.

## 4 Discussion

The nominal plans with planned scenarios, the results satisfied all dose regimens regardless of the angle configurations, as shown in **Table 2**. In addition, the differences in the *D*_95%_ of the RBE-weighted dose for the CTV were less than 0.1%. In the case of the rectum mean dose, although the oblique beam field produced differences in the RBE-weighted dose and the calculated LET value of the rectum compared to the parallel beam field, the dose regimen of the rectum was also satisfied regardless of the beam field pair. Hence, it is possible to apply a CIRT plan involving various angle pairs for prostate cancer.

However, in the dosimetric results considering perturbed scenarios, the differences in the characteristics of parallel beam pairs and oblique beam pairs were prominent. **Figure 5** shows the results of the characteristic analysis of angle beam field in each direction for the perturbed scenarios. As shown in **Figures 5E, F**, the plan results using a parallel beam pair were superior in the perturbed scenarios considering the position uncertainty in the lateral direction. In addition, as shown in **Figure 4**, this aspect was reflected in the narrowest RBE-weighted DVH distribution of angle pair C for the CTV. However, for the anterior direction of the RBE-weighted dose result in **Figure 5A**, the standard deviation of angle pair A was 7.9 times higher for angle pair C, and 2.1 times higher for angle pair E. In the case of the LET value, the standard deviation of angle pair C was 1.5 times higher than that of angle pair A and 1.1 times higher than that of angle pair E. These results show that oblique beam configurations such as angle pairs A and E were more robust than angle configuration C, which is a conventional angle pair, for set-up uncertainty in the anterior direction.

A high LET distribution area was located near the anterior portion of the prostate, as shown in **Figure 3**, which was consistent with the relatively high CTV *D*_95%_ of the LET value in the anterior direction of **Figure 5A**. Imai et al. discovered tumour volume and histological grade were related to local control (LC) and overall survival (OS) rate (28). Especially, the LET distribution in the tumour was low, and it was confirmed that the larger the tumour cell size, the lower the LC and OS (28). Masumoto et al. conducted a study on the correlation between local recurrence and intra tumour LET distribution, and found that no recurrence was found when the minimum dose-averaged LET value did not exceed 40 keV/μm (29). Hagiwara et al. founded that the higher the minimum dose-averaged LET value in gross tumour volume, the better the LC (30). Inaniwa et al. set the prostate LET prescription value as 80 keV/μm using LET optimization but the LET value in this study is reasonable (31).

The CIRT pass rate for the prostate can be further improved even if a relatively smaller margin is given when using vertical fields (32). In addition, as shown in **Figure 2**, even though robust optimization was performed considering the set-up uncertainty of the anterior direction (10.0 mm), to satisfy the rectum dose regimen, the dose distribution had a convex shape for rectum sparing in the posterior direction of the CTV. This dose distribution causes a particularly low acceptance ratio in perturbed scenarios.

For the rectum dose regimen, in the posterior perturbed scenario, an unsatisfactory result was achieved only for *V*_40Gy._ As for the satisfaction ratio according to the angle pair for the dose regimen of *V*_40Gy_ in the posterior direction, the pass rates in angle pairs C were 30.0%, B: 50.0%, angle pair A, D, and E: 100.0%, respectively. Based on these results, a more robust plan could be generated for the posterior direction using oblique beams, such as angle pairs A, D, and E, and this trend was consistent with the rectum *V*_50Gy_ and *V*_53Gy_. A spacer can be used to reduce rectal dose (33, 34). In particular, PT is effective in reducing the dose delivered to the OAR owing to patient positioning and range uncertainties, and as the distance between the prostate and rectum increases, dose escalation or hypofractionation is also possible (35, 36).

This study focused on the dosimetric effects on the prostate and rectum according to the angle configurations and referred to the protocol 9904 and 1002 (4, 19, 20). In this protocol, sufficient margin (i.e., 10 mm of Anterior and two laterals, 5 mm of cranial, caudal, and posterior) for the target considering range and position uncertainty were already reflected (21); The setup uncertainty of the prostate cancer in CIRT was about 2 mm (7, 37), and when the range uncertainty is assumed to be about 2% based on the maximum energy in our institution, the uncertainty is about ≤ 5.0 mm. Therefore, the robust evaluation corresponding to the margin set in this study considered both position and range uncertainty. However, research on the dosimetric effect according to uncertainty (range and/or location) and margin is needed. In particular, research that can suggest appropriate margins is possible through robust evaluation of PTV, CTV, and rectum according to uncertainty. In the near future, a study on the relationship between range uncertainty and margin using the clinical version of RayStation is planned to conduct a study on the appropriate margin for each direction.

## 5 Conclusions

In this study, dosimetric comparison according to the CIRT angle pair was performed for prostate cancer. By analysing the RBE-weighted dose as a function of angle pair, it was confirmed that a nominal plan, to which robust optimization was applied considering the set-up uncertainty, satisfied all dose regimens. For perturbed scenarios, considering the set-up margin and direction, oblique beam fields such as angle pairs 60°–300° and 120° – 240° were superior to parallel beam pairs (90°–270°) in rectum sparing. Therefore, it is worth implementing CIRT using an oblique angle pair in treating a patient with a large set-up uncertainty or when applying a parallel beam pair is difficult.

## Data availability statement

The original contributions presented in the study are included in the article/supplementary material. Further inquiries can be directed to the corresponding authors.

## Ethics statement

The studies involving human participants were reviewed and approved by The Institutional Review Board of Yonsei University Hospital. Written informed consent for participation was not required for this study in accordance with the national legislation and the institutional requirements.

## Author contributions

Conceptualization, MH, H-BS and WK; methodology, JK, C-HK; validation, C-HK and C-SH; formal analysis, H-BS, MH; investigation, SP, WK; writing—original draft preparation, H-BS, MH; writing—review and editing, MH, WK, C-SH; visualization, H-BS; supervision, MH. All authors contributed to the article and approved the submitted version.
